# Pharmacological inhibitors of the cystic fibrosis transmembrane conductance regulator exert off-target effects on epithelial cation channels

**DOI:** 10.1007/s00424-022-02758-9

**Published:** 2022-10-07

**Authors:** JinHeng Lin, Sean M. Gettings, Khaoula Talbi, Rainer Schreiber, Michael J. Taggart, Matthias Preller, Karl Kunzelmann, Mike Althaus, Michael A. Gray

**Affiliations:** 1 Biosciences Institute, Newcastle University, Newcastle upon Tyne, NE2 4HH UK; 2 Present Address: Department of Pharmacology, University of Oxford, Oxford, OX1 3QT UK; 3 School of Natural and Environmental Sciences, Newcastle University, Newcastle upon Tyne, NE1 7RU UK; 4 Physiological Institute, University of Regensburg, 93053 Regensburg, Germany; 5 Department of Natural Sciences/Institute for Functional Gene Analytics, Structural Biology Group, Bonn-Rhein-Sieg University of Applied Sciences, 53359 Rheinbach, Germany; 6 Present Address: Department of Natural Sciences /Institute for Functional Gene Analytics, Ion Transport Physiology Group, Bonn-Rhein-Sieg University of Applied Sciences, 53359 Rheinbach, Germany

**Keywords:** CFTR inhibitors, Off-target effects, Store-operated calcium entry, Orai1, ENaC, In silico modelling

## Abstract

The cystic fibrosis transmembrane conductance regulator (CFTR) anion channel and the epithelial Na^+^ channel (ENaC) play essential roles in transepithelial ion and fluid transport in numerous epithelial tissues. Inhibitors of both channels have been important tools for defining their physiological role in vitro. However, two commonly used CFTR inhibitors, CFTR_inh_-172 and GlyH-101, also inhibit non-CFTR anion channels, indicating they are not CFTR specific. However, the potential off-target effects of these inhibitors on epithelial cation channels has to date not been addressed. Here, we show that both CFTR blockers, at concentrations routinely employed by many researchers, caused a significant inhibition of store-operated calcium entry (SOCE) that was time-dependent, poorly reversible and independent of CFTR. Patch clamp experiments showed that both CFTR_inh_-172 and GlyH-101 caused a significant block of Orai1-mediated whole cell currents, establishing that they likely reduce SOCE via modulation of this Ca^2+^ release-activated Ca^2+^ (CRAC) channel. In addition to off-target effects on calcium channels, both inhibitors significantly reduced human αβγ-ENaC-mediated currents after heterologous expression in *Xenopus* oocytes, but had differential effects on δβγ-ENaC function. Molecular docking identified two putative binding sites in the extracellular domain of ENaC for both CFTR blockers. Together, our results indicate that caution is needed when using these two CFTR inhibitors to dissect the role of CFTR, and potentially ENaC, in physiological processes.

**Supplementary Information:**

The online version contains supplementary material available at 10.1007/s00424-022-02758-9.

## Introduction

CFTR and ENaC play essential roles in ion and fluid transport in numerous epithelial tissues, dysfunction of which leads to diseases such as cystic fibrosis (CF), secretory diarrhoea and kidney disease [1]. Two drugs assumed to be relatively selective CFTR inhibitors, CFTR_inh_-172 and GlyH-101 [2, 3], have been important tools for defining the role of CFTR in transepithelial ion transport in vitro*.* Despite being widely used, these agents are not solely selective for CFTR, as they inhibit other types of Cl^−^ channels and even affect mitochondrial function [4–6]. Moreover, GlyH-101 also exhibited off-target effects on voltage-gated Ca^2+^ and K^+^ channels in isolated ventricular myocytes [7]. Together, these studies suggest that these CFTR inhibitors may have more widespread off-target effects than originally appreciated. However, the potential modulatory effects of these inhibitors on epithelial cation channels to date have not been investigated. This issue is important because CFTR has been shown to modulate Ca^2+^ signalling in epithelial cells via multiple mechanisms, which ultimately leads to raised cytosolic Ca^2+^ levels and cellular dysfunction [8]. These mechanisms include altered release of Ca^2+^ from ER stores, as well as enhanced Ca^2+^ influx through Ca^2+^-permeable channels, such as the store-operated calcium entry (SOCE) channel Orai1 and transient receptor potential (TRP) channels [9]. Disrupted Ca^2+^ signalling due to the absence or malfunction of CFTR can also alter mitochondrial function, which can lead to cell injury, and ultimately activate apoptosis or necrosis [8]. In addition to effects on Ca^2+^ signalling, CFTR dysfunction also stimulates epithelial Na^+^ transport into cells, via increased ENaC activity [10]. However, the exact molecular mechanisms involved in either of these actions of CFTR on epithelial cation transport are incompletely understood. Significantly, some of the published studies implicating CFTR in the regulation of epithelial Ca^2+^ homeostasis have employed CFTR inhibitors to validate the role of this anion channel in Ca^2+^ signalling [11–15]. We therefore felt it was important to investigate if these CFTR inhibitors had modulatory effects on Ca^2+^-entry channels, both in the presence and absence of functional CFTR, as well as investigate any off-target effects directly on ENaC function. Here, we demonstrate for the first time that CFTR blockers cause a significant inhibition of SOCE, via block of the Orai1 Ca^2+^ influx channel, as well isoform-specific modulation of ENaC. Our results therefore highlight that care needs to be taken when assigning a specific physiological, or pathophysiological, role for CFTR in epithelial function based on the results using these two CFTR inhibitors.

## Materials and methods

### Cell culture

Calu-3 cells were cultured in Eagle’s minimum essential medium; HEK293T cells were cultured in Dulbecco’s modified Eagle’s medium (DMEM) or in DMEM low glucose medium. All culture media were supplemented with 100 U/ml penicillin, 100 μg/ml streptomycin, 2 mM L-glutamine, 1% non-essential amino-acid (NEAA) and 10% foetal bovine serum (FBS).

### Transfection of cells

HEK293T cells were transfected using Lipofectamine 3000 (as per manufacturer’s instructions), with a bicistronic IRES plasmid vector encoding hOrai1 and CD8, and a plasmid encoding hStromal Interacting Molecule 1 (STIM1), at a ratio of 1/10, respectively. Control cells were mock transfected with an empty pcDNA3.1 vector (mock) and CD8 [16]. Transfected cells were visually detected by binding of anti-CD8 labelled beads [17]. All experiments were performed 48 h after transfection.

### Real‐time quantitative PCR

Calu-3 and HEK293 cells were lysed for RNA extraction using the RNeasy Mini Kit (Qiagen). Reverse transcription and real-time qPCR was performed as previously described [18]. PCR primers (CFTR (Forward – AGGAGGCAGTCTGTCCTGAA; Reverse – CACTGCTGGTATGCTCTCCA), GAPDH (Forward – TGCACCACCAACTGCTTAGC; Reverse – GGCATGGACTGTGGTCATGAG)) were purchased from Integrated DNA Technologies (Leuven, Belgium).

### Western blotting

Calu-3 and HEK293 cells were lysed in ice-cold RIPA buffer, and Western blot was performed as previously described [18]. A total of 30 μg of protein were loaded onto an 8% SDS-PAGE gel for electrophoresis, and protein was transferred onto a PVDF membrane (0.1 A constant for 60 min). Membranes were blocked with 5% milk (1 h at room temperature (RT)), then incubated with the primary (1:1000, overnight at 4 °C) and secondary antibody (1:5000, 1 h at RT). Primary antibody was CFFT-596, raised in mouse, from the Cystic Fibrosis Foundation’s Antibody Distribution Program.

### Intracellular Ca^2+^ measurements

Intracellular Ca^2+^ ([Ca^2+^]_i_) was measured using the Ca^2+^-sensitive fluorescent dye fura-2-acetoxymethylester (fura-2-AM; Thermo Fisher Scientific) using cells grown on 25-mm glass coverslips as previously described [19]. Fura-2 was excited alternately at 340 nm and 380 nm for 250 ms, and emitted light captured at 510 nm; the 340/380 emission ratio (F340/380) of fura-2 fluorescence reflects [Ca^2+^]_i_ levels. Store-operated channels (SOCs) channels were activated by depleting [Ca^2+^]_i_ stores with 200 nM thapsigargin (Tg), in nominally Ca^2+^-free conditions, and then SOCE measured by tracking changes in [Ca^2+^]_i_ using a repeated extracellular Ca^2+^ addback protocol, alternating between a Ca^2+^-free solution and one containing 1 mM Ca^2+^. The composition of the HEPES-buffered solution was (in mM): 130 NaCl, 5 KCl, 1 CaCl_2_, 1 MgCl_2_, 10 4-(2-hydroxyethyl)piperazine-1-ethanesulfonic acid sodium salt (NaHEPES), 10 D-glucose, (pH 7.4). For the nominally Ca^2+^-free solution, CaCl_2_ was omitted and replaced with 1 mM ethylene glycol-bis(2-aminoethylether)-N,N,N,N-tetraacetic acid (EGTA). SOCE amplitude was calculated as the difference between baseline F340/380 ratio (average of 30 s data) before a response and maximum peak value (average of 1–2 s of data) and calculated as peak–baseline. SOCE rate was calculated by linear regression to the initial, steepest, portion of the [Ca^2+^]_i_ increase within the first few minutes after calcium addback.

### Whole cell patch clamp

Cells were patch clamped after growing them on coated glass coverslips for 2 days. Patch clamp experiments were performed in the fast whole-cell configuration. Patch pipettes had an input resistance of 3–6 MΩ, when filled with a solution containing in (mM) KCl 30, K^+^-gluconate 95, NaH_2_PO_4_ 1.2, Na_2_HPO_4_ 4.8, EGTA 1, Ca^2+^-gluconate 0.758, MgCl_2_ 1.034, D-glucose 5, ATP 3, pH 7.2 and Ca^2+^ activity 0.1 μM. The bath was perfused continuously with a standard bicarbonate-free Ringer’s solution, composed of (in mM) NaCl 145, KH_2_PO_4_ 0.4, K_2_ HPO_4_ 1.6, MgCl_2_ 1, Ca-gluconate 1.3, glucose 5, pH 7.4) at a rate of 4 mL/min. The access conductance was continuously measured and was 40–100 nS. Currents were recorded with an EPC-7 patch clamp amplifier (List Medical Electronics, Germany), the LIH1600 interface and PULSE software (HEKA, Germany) as well as Chart software (AD-Instruments, Germany). Data were stored continuously on a computer hard disc and analysed using PULSE software. In regular intervals, membrane voltages (*V*c) were clamped in steps of 20 mV from − 100 to + 100 mV relative to resting potential.

### Plasmids and cRNA synthesis

Coding DNA sequences for human ENaC subunits (α, β, γ and δ) were present in the pTNT expression vector (Promega, UK). Plasmids were linearised with FastDigest *Bam*H1 (ThermoFisher Scientifc, UK) and capped cRNA was generated using the T7 Ribo-MAX large-scale RNA production system (Promega) following manufacturer’s instructions. ENaC-subunits were diluted with diethyl pyrocarbonate (DEPC)-treated water to a final concentration of 10 ng/μl per ENaC subunit and combined to αβγ-ENaC or δβγ-ENaC cRNA solutions.

### Heterologous expression of ENaC in Xenopus oocytes

*Xenopus laevis* ovaries were purchased from the European Xenopus Resource Centre (EXRC, Portsmouth, UK). The experimental procedures were approved by the Newcastle University Animal Welfare and Ethical Review Body (AWERB, project ID 630). Oocytes were isolated by incubation in collagenase and Ca^2+^-free solution as previously described [20]. Stage V/VI oocytes were injected (Nanoject, Drummond Scientific, Broomall, USA) with 13.8 nl of αβγ-ENaC or δβγ-ENaC cRNA, or DEPC-treated water for control experiments. Injected oocytes were incubated for 24–48 h at 16 °C in a low sodium oocyte Ringer solution containing (in mM) 80 N-methyl-D-glucamine, 10 NaCl, 1 KCl, 2 CaCl_2_, 2.5 sodium pyruvate, 5 HEPES (pH 7.4) and supplemented with 20 μg/ml gentamycin. All procedures for oocyte isolation, expression of human ENaC subunits (α, β, γ and δ) were exactly as previously described [20].

### Two-electrode voltage-clamp recordings

Oocytes were placed in a recording chamber filled with oocyte ringer solution (ORS), containing (in mM) 90 NaCl, 1 KCl, 2 CaCl_2_ and 5 HEPES (pH 7.4). Chloride-coated silver wires were inserted into microelectrodes, which were pulled from borosilicate glass capillaries and filled with 1 M KCl. For reference electrodes, chloride-coated silver wires were placed into 1 M KCl/agar (3%) bridges. Oocytes were clamped to − 60 mV with an OC725B/C oocyte voltage clamp amplifier (Warner Instruments, Hamden, USA) and transmembrane currents (I_M_) were filtered at 1 kHz (Warner Instruments) and recorded on a strip chart recorder (Kipp&Zonen, Delft, The Netherlands). Experiments were performed at RT under continuous superfusion (5 ml/min) of oocytes with ORS.

### Molecular docking

Docking experiments were carried out using the cryo-EM structure of ENaC (pdb: 6bqn) and the closed and open structures of Orai1 (pdb: 4hkr and 7kr5). Missing loops in the proteins and side chains were added via comparative structure modelling with Modeller [21]. The three-dimensional structures of CFTR_inh_-172, GlyH-101 and amiloride were obtained from PubChem. AutodockTools [22] was used to prepare the protein and ligand structures. Initial blind docking with subsequent targeted docking for individual binding pockets in ENaC were performed using Autodock Vina [23], and an exhaustiveness of 56.

### Chemicals

All reagents, including amiloride hydrochloride hydrate, were purchased from Sigma-Aldrich (UK, Germany) unless otherwise specified. Thapsigargin (Tg), CFTR_inh_-172, GlyH-101 and cyclopiazonic acid (CPA) were purchased from Tocris (Abington, UK) or (Deisenhofen Germany). Dimethyl sulfoxide (DMSO) from ThermoFisher Scientific (Gloucester, UK). For all experiments, stock solutions of 20 mM CFTRinh-172, 100 mM GlyH-101 and 100 mM amiloride were prepared in DMSO. Stock solutions were diluted in bathing solutions and DMSO concentrations were kept at, or below, 0.2%.

### Statistics

Statistical analysis was performed using GraphPad Prism 8 (GraphPad Software, San Diego, USA), with statistical significance indicated by an alpha value of *p* < 0.05. The type of analysis for each dataset, including post-hoc analysis, is indicated in the respective figure legend.

## Results

### CFTR_inh_-172 causes a time-dependent reduction in SOCE in Calu-3 cells

**Fig. 1:**
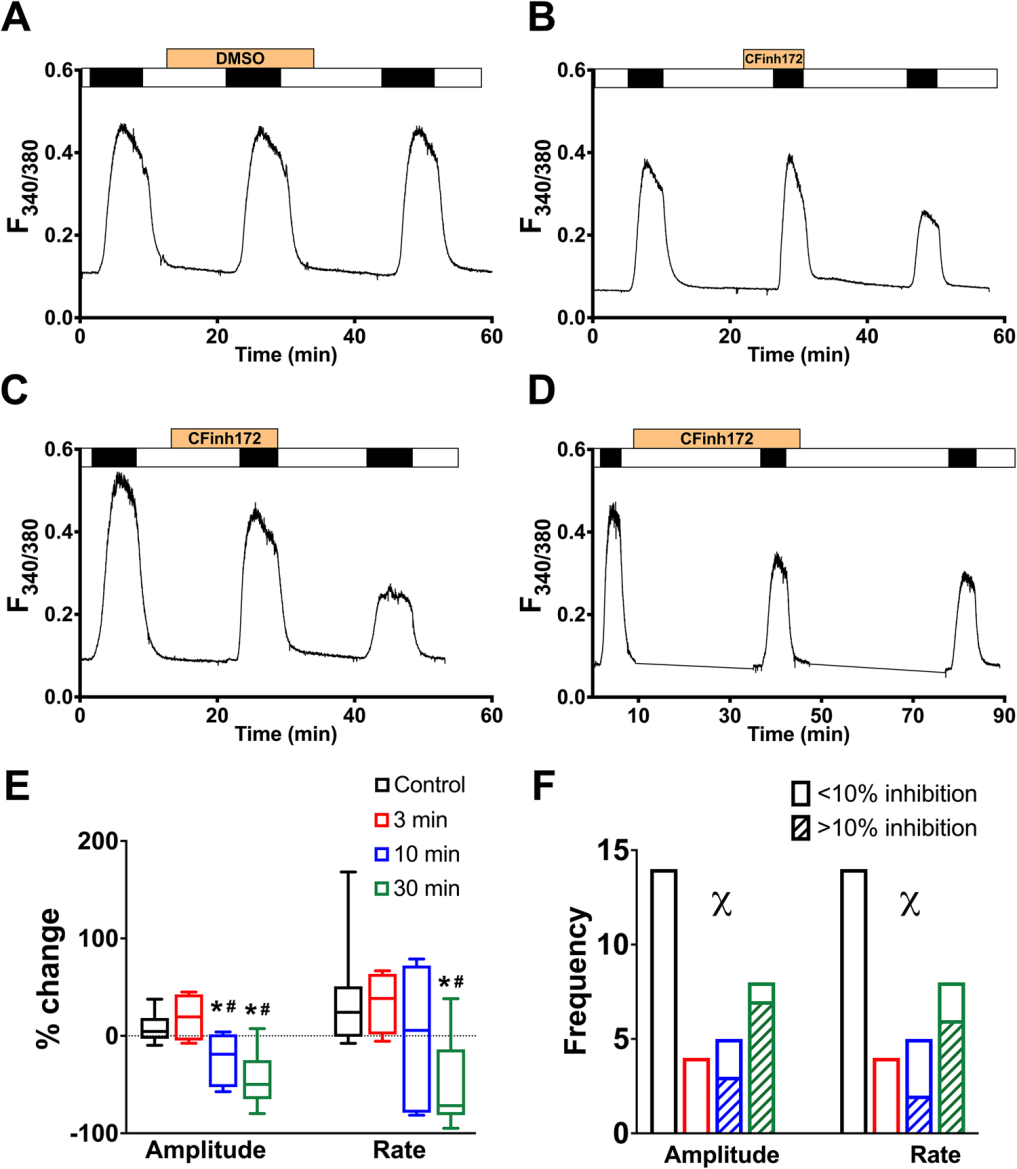
Time-dependent inhibition of SOCE by CFTR_inh_-172 in Calu-3 cells. **A**–**D** Representative Ca^2+^ fluorimetry traces tracking changes in [Ca^2+^]_i_ following a Tg-activated, repeated Ca^2+^ addback protocol. Cells were exposed to the vehicle DMSO (**A**), or 20 µM CFTR_inh_-172 (CFinh172, B-D), in a nominally Ca^2+^ free solution for 3 (**B**), 10 (**C**), or 30 (**D**) min before the second SOCE was induced by adding back 1 mM Ca.^2+^ (black bars). The agents were removed from the perfusing solution for 15 (**B**, **C**) or 30 (**D**) min before the third SOCE was activated. (**E**) Box and whiskers summary of percentage change in SOCE peak amplitude and rate, from SOCE #1 to SOCE #2, following DMSO (control) or CFTR_inh_-172 treatment of different durations. One-way ANOVA with Holm-Sidak multiple comparisons tests was performed across the four groups. Boxes represent median ± 25th/75th percentiles, while whiskers represent minimum/maximum. * = *p* < 0.05 vs. control; # = *p* < 0.05 vs. 3 min. **F** Frequency of > 10% inhibition of SOCE amplitude and rate by DMSO or CFTR_inh_-172 treatment of different durations. Percentage inhibition was calculated as percentage of SOCE #2 amplitude/rate over that of SOCE #1, and the frequency of experiments with over and under 10% inhibition was tallied. A chi-square test was performed across the four groups. *χ* = *p* < 0.05 for chi-square test. *n* = 4–14

Calu-3 cells are a human airway-derived cell line that expresses high levels of functional CFTR [24], which can be effectively inhibited by application of 20 µM CFTR_inh_-172 [2, 6, 25], which was confirmed by our own studies (Supplementary Fig. 1). To investigate the effect of CFTR_inh_-172 on SOCE in these cells, intracellular Ca^2+^ stores were first passively depleted with 200 nM thapsigargin (a SERCA pump inhibitor), in nominally Ca^2+^-free conditions, to activate store-operated channels (SOC). Figure 1 shows that the amplitude of SOCE, measured using a repeated Ca^2+^-addback protocol (see Material and Methods), was significantly reduced by pre-treating cells with 20 µM CFTR_inh_-172 in a time-dependent manner (24.2 ± 12.3% decrease after 10 min, *p* = 0.026 and 44.2 ± 10.0% after 30-min treatment, p < 0.0001 versus vehicle-treated cells). The initial rate of SOCE was also reduced for the 30 min pre-treated group (52.0 ± 16.4%, *p* = 0.004). Overall, the frequency of observing a greater than 10% inhibition in both amplitude and rate of SOCE by CFTR_inh_-172 was time-dependent (Fig. 1F; *p* < 0.05, chi-square test). The inhibitory effect of CFTR_inh_-172 was essentially irreversible up to 30 min of washout of the compound (Fig. 1D).

### CFTR_inh_-172 and GlyH-101 inhibit SOCE in epithelial cells that do not express CFTR

**Fig. 2:**
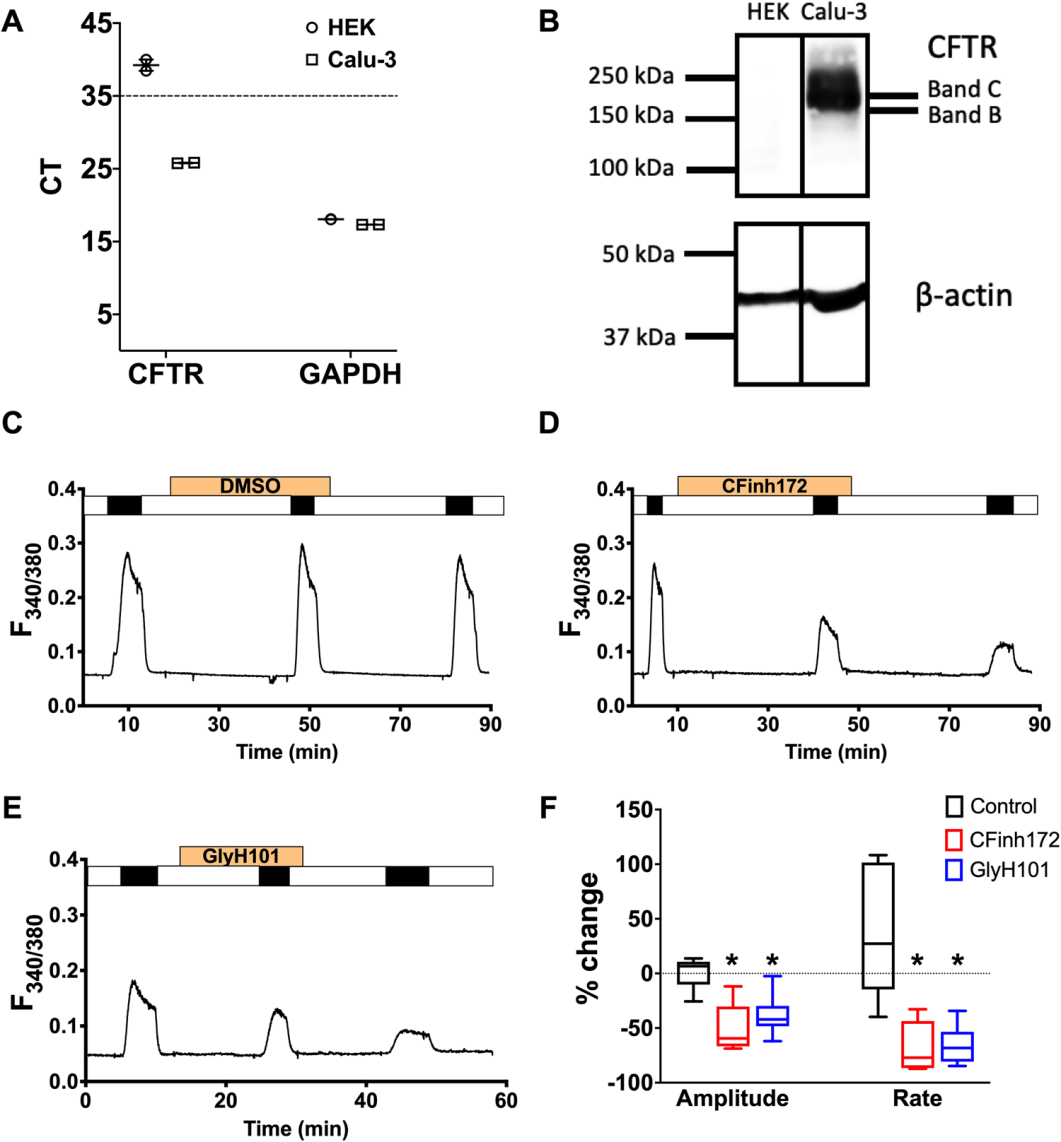
Distinct CFTR inhibitors reduce SOCE in HEK293T cells. **A**, **B** HEK293T cells do not express CFTR. **A** Raw CT values for amplification of CFTR, and the housekeeping gene GAPDH, in Calu-3 and HEK293T cell samples. Each sample was run in duplicate. **B** Western blot image of Calu-3 and HEK293T samples probed for CFTR expression (Band C ~ 180 kDa, fully glycosylated CFTR). **C**–**E** Representative Ca^2+^ fluorimetry traces tracking changes in [Ca^2+^]_i_ following a Tg-activated, repeated Ca.^2+^ addback protocol, with pre-treatment of DMSO (**C**), 20 µM CFTR_inh_-172 for 30 min (**D**) or 10 µM GlyH-101 for 10 min (**E**) before the second SOCE was activated. **F** Box and whiskers summary of percentage change in SOCE peak amplitude and rate, from SOCE #1 to SOCE #2, following treatment of DMSO (control) or the CFTR inhibitors. One-way ANOVA with Holm-Sidak multiple comparisons tests was performed across the three groups. Boxes represent median ± 25th/75th percentiles, while whiskers represent minimum/maximum. * = *p* < 0.05 vs. control. *n* = 5–6

To investigate if the reduction in SOCE by CFTR_inh_-172 was dependent on CFTR, we repeated the Ca^2+^-addback protocol on HEK293T cells, which do not express CFTR (Fig. 2A, B). We also tested another well-known CFTR inhibitor, GlyH-101, on SOCE. Figure 2C–F show that both CFTR inhibitors reduced SOCE amplitude and rate. Pre-treatment of HEK293 cells with 20 µM CFTR_inh_-172 for 30 min or 10 µM GlyH-101 for 10 min, inhibited the amplitude (50.7 ± 10.3%, *p* = 0.003 and 38.5 ± 8.0%, *p* = 0.001, respectively, vs. vehicle-treated) and rate (67.5 ± 10.5%, *p* = 0.002 and 65.7 ± 7.3%, *p* = 0.002, respectively, vs. vehicle-treated) of SOCE. These results clearly show that the inhibitory effect of these two putative CFTR inhibitors is not dependent on CFTR expression.

### CFTR_inh_-172 and GlyH-101 inhibit Orai1/Stim1-mediated currents in transfected HEK293T cells

**Fig. 3:**
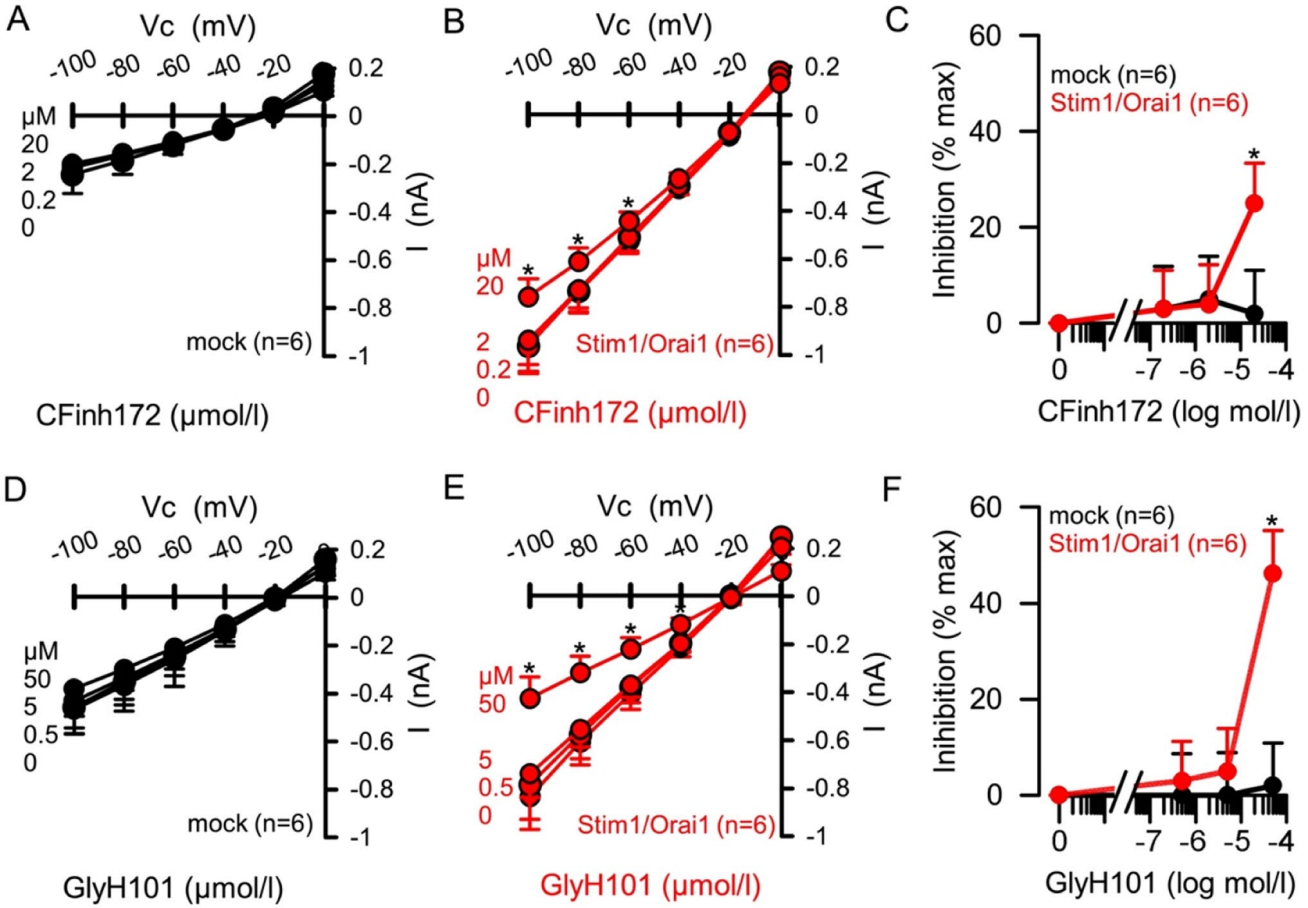
CFTR_inh_-172 and GlyH-101 inhibit Orai1/Stim1-mediated whole cell currents in HEK293T cells. **A**, **B**, **D**, **E** Current–voltage relationships for whole cell currents measured in cells pre-exposed to CPA (10 µM) to activate Orai1 channels, followed by CFTR inhibitors. The inhibitors were present for 3 min at each concentration tested. Cells were mock-transfected (black symbols) or overexpressed Orai1/Stim1 (red symbols). Increasing concentrations of CFTR_inh_-172 (CFinh172, 0.2, 2, 20 µM) or GlyH-101 (GlyH101, 0.5, 5, 50 µM) were applied in the continuous presence of CPA. Application of the highest concentration of CFTR_inh_-172 (20 µM) or GlyH-101 (50 µM) significantly inhibited the inward currents, but had no effect in mock-transfected cells. **C**, **F** Summary of the concentration-dependent inhibition of inward currents by CFTR_inh_-172 or GlyH-101. * = *p* < 0.05 vs. control (mock). Un-paired *t*-test

The results in Fig. 2 clearly show that both CFTR inhibitors reduced SOCE activity in a CFTR-independent manner. To gain insight into the mode of action of these two inhibitors, we investigated their effect on Orai1-mediated whole cell currents in HEK293T cells, using the patch clamp technique. Ca^2+^ release-activated Ca^2+^ (CRAC; CRACM) channels responsible for SOCE are small and difficult to detect [26, 27]. While SOCE often can be measured by fluorimetry, CRAC-mediated currents produced by endogenous Orai1/Stim1 channels cannot be resolved in the presence of physiological ion concentrations. In order to amplify CRAC, we used cells overexpressing Orai1 and Stim1 to aid measurements of CRAC currents [26], and activated them by store depletion using CPA, another SERCA pump inhibitor. CPA induced a small, but not significant, current in mock-transfected cells, which was larger in Orai1/Stim1-overexpressing cells. Figure 3A, B, D and E show current/voltage relationships of the inward currents after stimulation with CPA, and in the presence of different concentrations of CFTR_inh_-172 and GlyH-101. At maximal concentrations of both inhibitors, inward currents were significantly reduced in overexpressing cells, but not in non-transfected cells. Concentration-dependent inhibition of inward currents was determined by setting the inward currents at Vc =  − 100 mV, in the absence of inhibitors, to 100%, and relating this to the inward currents in the presence of different inhibitor concentrations (Fig. 3C, F). While lower concentrations of each inhibitor did not affect inward currents, maximal concentrations of CFTR_inh_-172 and GlyH-101 clearly inhibited CPA-activated inward currents in Orai1/Stim1 overexpressing cells, suggesting block of CRAC by inhibitors of CFTR. We confirmed that both CFTR inhibitors significantly inhibited CFTR-dependent whole cell currents in transfected HEK cells (Supplementary Fig. 2).

Molecular docking of CFTR_inh_-172 and GlyH-101 to the structures of Orai1 in the closed and open states (pdb: 4hkr [28] and 7kr5 [29]) did not result in a reliable prediction of the binding site. All of the possible, detected sites in the available Orai1 structures included larger unresolved areas of the structure or led to low predicted binding affinities. The best predicted binding site between the transmembrane helices TM1 and TM2, as well as TM1 of the adjacent protein chain, which was obtained in the closed Orai1 structure, comprises at least 16 amino acid residues, which are missing in the crystal structure, and is hence unreliable, as also reported for the docking of AnCoA4 to Orai1 [30]. This region is completely missing in the recent cryo-EM structure of Orai1 in the open state.

### Both CFTR_inh_-172 and GlyH-101 affect ENaC-mediated currents in Xenopus oocytes

**Fig. 4:**
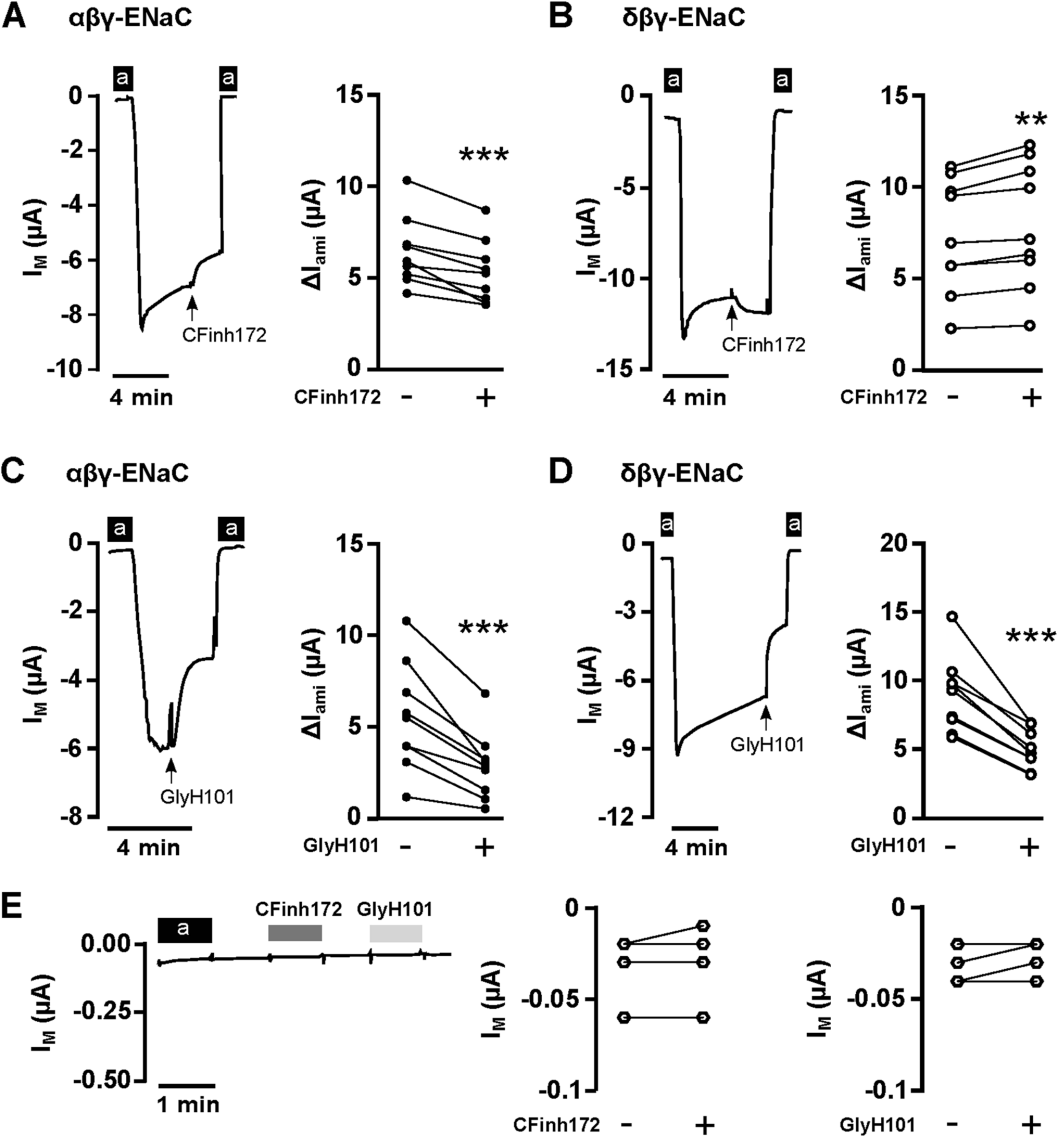
CFTR_inh_-172 and GlyH-101 affect ENaC currents in *Xenopus* oocytes. **A** Left panel: representative current trace of a human αβγ-ENaC expressing oocyte. The application of amiloride (100 µM; ‘a’) is represented by the black bars and was used to determine amiloride-sensitive fractions of *I*_M_ (*ΔI*_ami_; right panel), before and after application of CFTR_inh_172 (20 µM; CFinh172). *n* = 9. **B** Similar experiments as shown in panel **A**, with human δβγ-ENaC expressing oocytes (*n* = 9). **C**/**D** Similar experiments as shown in panels **A**/**B**, where *ΔI*_ami_ were determined before and after application of GlyH-101 (GlyH101, 10 µM) *n* = 9 for both conditions. Student’s paired *t*-test was employed for all statistical analyses, with ** indicating *p* < 0.01 and *** indicating *p* < 0.001. **E** Left panel: Representative current trace of a water-injected control oocyte. The application of amiloride (100 µM; ‘a’) is represented by the black bars, the application of CFTR inhibitors by grey bars. Neither of the CFTR-inhibitors had any significant effect on transmembrane currents (I_M_). *n* = 6. Wilcoxon matched-pairs signed rank test was employed for statistical analyses

To investigate if the inhibitory effect of the two CFTR blockers was restricted to Ca^2+^-permeable channels, we studied their effect on ENaC-mediated sodium currents in *Xenopus* oocytes expressing either the ‘classical’ αβγ-ENaC, or oocytes expressing δβγ-ENaC. Oocytes are an excellent model system to study the effect of compounds on specific channel isoforms, and for ENaC, this is convenient given there are two isoforms and there is no suitable model for studying δβγ-ENaC. The ENaC blocker amiloride [31] was used to determine amiloride-sensitive (*ΔI*_ami_), i.e. ENaC-mediated, currents before and after application of the CFTR blockers (Fig. 4). In *Xenopus* oocytes expressing αβγ-ENaC, 20 µM CFTR_inh_-172 significantly inhibited *ΔI*_ami_ by 17.45 ± 3.02% from 6.42 ± 0.62 µA to 5.32 ± 0.58 µA (*n* = 9; *p* = 0.0004; Student’s paired *t*-test; Fig. 4A). By contrast, ΔI_ami_ in δβγ-ENaC expressing oocytes significantly increased by 8.2 ± 1.13% from 7.32 ± 1.04 µA to 7.93 ± 1.15 µA in response to CFTR_inh_-172 (*n* = 9; *p* = 0.0019; Student’s paired *t*-test; Fig. 4B). GlyH-101 (10 µM) had a strong inhibitory effect on both ENaC isoforms. In *Xenopus* oocytes expressing αβγ-ENaC, ΔI_ami_ decreased by 49.6 ± 3.96% from 5.52 ± 0.98 µA to 2.87 ± 0.62 µA (*n* = 9; *p* = 0.0005; paired Student’s *t*-test; Fig. 4C). The ΔI_ami_ in δβγ-ENaC expressing oocytes decreased by 43.63 ± 2.3% from 8.99 ± 0.91 µA to 5.02 ± 0.47 µA (*n* = 9; *p* < 0.000; paired Student’s *t*-test; Fig. 4D). Water-injected control oocytes did not respond to amiloride, CFTR_inh_-172, or GlyH-101 (Fig. 4E). The transmembrane currents (*I*_M_) of water-injected control oocytes were − 0.032 ± 0.006 µA before, and − 0.03 ± 0.007 µA after exposure to CFTR_inh_-172 (*n* = 6, *p* > 0.999; Wilcoxon matched-pairs signed rank test), and − 0.032 ± 0.004 µA before, and − 0.027 ± 0.003 µA after exposure to GlyH-101 (*n* = 6, *p* = 0.25; Wilcoxon matched-pairs signed rank test).

**Fig. 5:**
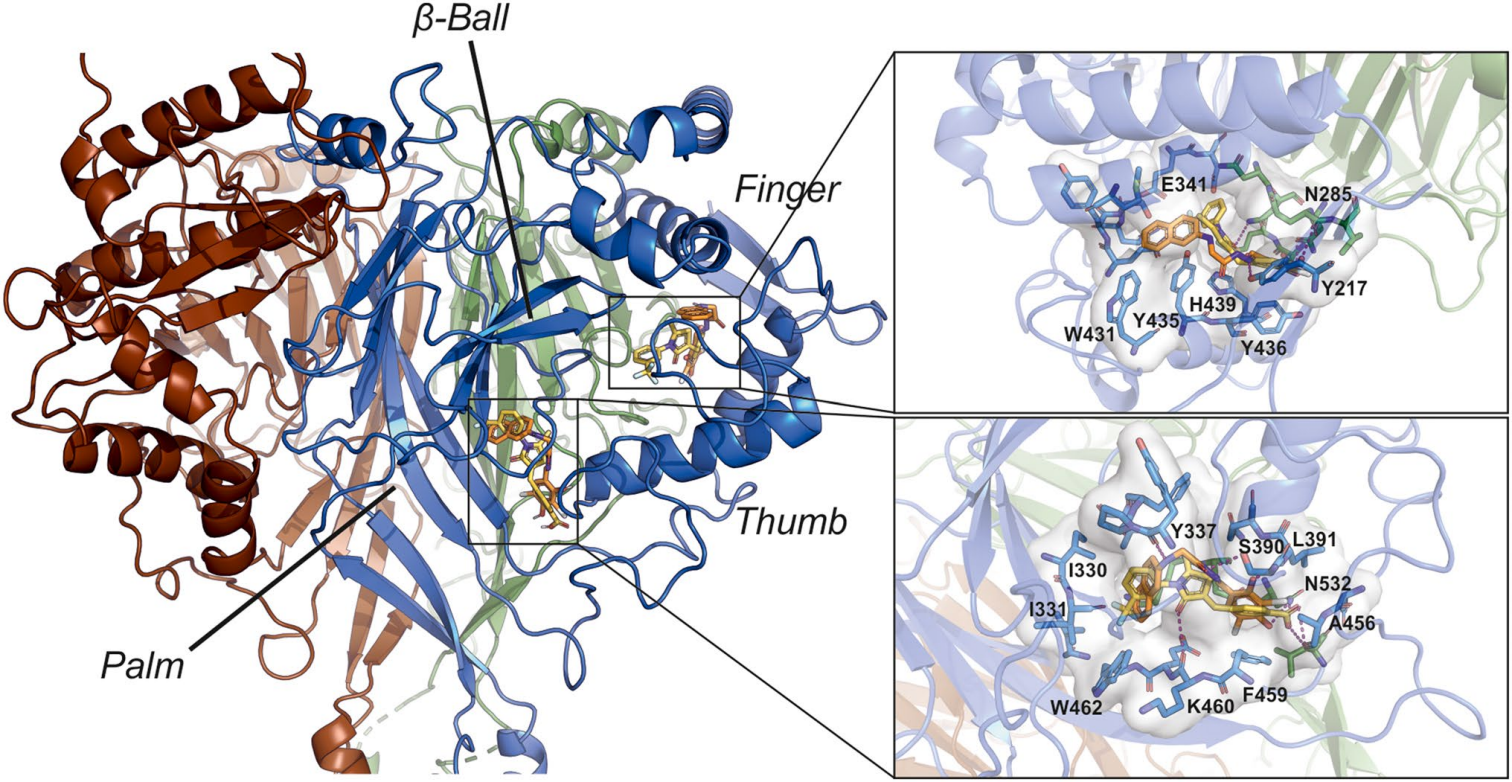
Putative CFTR-inhibitor binding sites in human ENaC. Molecular docking experiments identified two potential binding pockets for CFTR_inh_-172 (yellow) and GlyH-101 (orange) in the human α-ENaC structure. α-ENaC is shown in blue, γ-ENaC in brown and β-ENaC in green. The structure of each ENaC subunit represents a clenched hand holding a ball of β-sheets [32]. One binding pocket is located at the ‘finger’/’ thumb’ domain interface, the second binding pocket is located between the ‘β-ball’ and ‘palm’ domains

Our results suggest that both inhibitors bind to ENaC to inhibit function. We used molecular docking experiments to predict the putative binding site of the two compounds to the structure of human αβγ-ENaC (pdb: 6bqn; [32]). Initial blind docking allowed scanning for possible binding pockets in the structure. As a reference, we also docked amiloride into the same structure. For all three compounds, we identified binding of the ligands only at binding sites in the extracellular domain. In a series of subsequent targeted docking experiments with the individual binding sites as search areas, for both blockers, two putative binding sites in the ENaC α-subunit were identified, which yielded high affinity binding of CFTR_inh_-172 and GlyH-101 (Fig. 5A). The first binding site is located near the finger-thumb interface of ENaC, in close proximity to the reported extracellular motif WYKLHY (WYRFHY in rat), which was shown to be involved in amiloride binding [33]. Both blockers bind mostly through hydrophobic interactions to the nonpolar binding site, including residues Tyr-217, Tyr-435, Tyr-436 and His-439. Polar contacts were formed between GlyH-101 and amino acids Tyr-217 and Asn-285. The second putative binding pocket is located at the interface between two ENaC subdomains (between the ‘β-ball’ and ‘palm’). Binding of CFTR_inh_-172 was stabilised by hydrophobic and aromatic interactions with amino acids Ile-330, Ile-331, Val-343, Leu-391, Ala-456 Phe-459 and Trp-462, as well as hydrogen bonds with residues Ala-456, Lys-460, and Asn-532 (Fig. 5). Similarly, the best binding of GlyH-101 was also found at the same site in ENaC. The aromatic rings of GlyH-101 were accommodated at the same areas in the binding pocket and interact with the same hydrophobic residues as CFTR_inh_-172. Hydrogen bonds of GlyH-101 were formed with amino acids Ser-390, Leu-391, Gln-392 and Asn-532 (Fig. 5).

## Discussion

Our results show that the putative specific CFTR inhibitors, CFTR_inh_-172 and GlyH-101, exerted CFTR-independent inhibition of SOCE in human epithelial cell lines, likely through inhibition of Orai1-mediated Ca^2+^ entry. In *Xenopus* oocytes, both inhibitors also significantly reduced ENaC-mediated currents differentially based on ENaC subunit composition. Thus, our results provide strong evidence for off-target effects of these CFTR inhibitors on two distinct classes of physiologically important epithelial cation channels.

The specificity of both CFTR_inh_-172 and GlyH-101 has previously been questioned. Melis et al. [6] showed that, in addition to CFTR, these agents also inhibited volume-sensitive Cl^−^ channels, and GlyH-101 inhibited Ca^2+^-activated Cl^−^ channels. Melis et al. [6] also demonstrated that these agents were also cytotoxic when incubated with cells for 24 h (> 5 and 10 µM for CFTR_inh_-172 and GlyH-101, respectively). Moreover, GlyH-101, but not CFTR_inh_-172, inhibited SLC26A9, which facilitates Cl^−^ transport in epithelial cells [4]. However, our study is the first to report off-target effects of CFTR_inh_-172 and GlyH-101 on SOCE in epithelial cells at concentrations used by others to inhibit CFTR. In our hands, 20 µM CFTR_inh_-172 significantly inhibited SOCE in both patch clamp and Ca^2+^ fluorimetry studies, whereas GlyH-101 inhibited SOCE in Ca^2+^ experiments at 10 µM, but in patch clamp experiments, while 5 µM did not, 50 µM did. The initial screening study indicated that CFTR_inh_-172 completely blocked CFTR currents at 5 µM [2], and the working concentration commonly used by others range from 10 [11, 13, 34, 35], 20 [25, 36–38] and up to 100 µM [39]. On the other hand, GlyH-101 is commonly used between 10 [40] and 50 µM [4, 41]. Therefore, the concentrations employed in the present study are within the range used by others, including 50 µM GlyH-101, despite potential cytotoxicity issues [6], and therefore, are relevant to the field in general.

In addition, we showed that CFTR_inh_-172 exerted a time-dependent and poorly reversible inhibition of SOCE in both Calu-3 and HEK293T cells. Using Calu-3 cells, we established that a short (3 min) pre-exposure to CFTR_inh_-172 had no effect on the subsequent magnitude or rate of SOCE, but longer exposures did. Because a short exposure to CFTR_inh_-172 is generally sufficient to inhibit CFTR in epithelial cells [2], this result suggests that inhibiting CFTR in Calu-3 cells does not directly affect SOCE. Rather, our data suggests that the inhibition of SOCE is an off-target effect on Ca^2+^ influx itself, which does not require CFTR. This was confirmed in HEK293T cells that do not express detectable levels of CFTR, but which demonstrated a similar level of block of SOCE by CFTR_inh_-172. In most epithelial cells, store depletion leads to the activation of Orai1 channels, a process initiated by ER-localised STIM1 oligomerisation, followed by STIM1 translocation and subsequent Orai1 channel opening [42]. To investigate if Orai1/STIM1 were involved in the inhibitory effects of CFTR_inh_-172 on SOCE, the two proteins were overexpressed in HEK293T cells. This was necessary because endogenous levels of Orai1-Ca^2+^ currents are difficult to detect electrophysiologically [26]. Our results showed a clear concentration-dependent reduction of Orai1-mediated currents by both CFTR inhibitors, confirming that they inhibit Orai1 channels. Both these CFTR inhibitors share chemical features with the Orai1 inhibitor AnCoA4 [30], including a central aromatic system and at least two functional groups with hydrogen bond acceptor properties, at distances of 2.5 to 3.5 Å from the aromatic system, which might suggest a similar and direct binding of the two blockers to the Orai1 channel. Unfortunately, missing areas in the two higher-resolution structures of Orai1 did not allow us to reliably identify any binding sites for CFTR_inh_-172 and GlyH-101 in the Orai1 channel.

An interesting aspect of the inhibitory effect of CFTR_inh_-172 on SOCE was the time lag to achieve inhibition. In the original study that identified the compound [2], it irreversibly inhibited CFTR current within 2 min of application. However, as shown here, a 3 min and even a 10-min exposure to CFTR_inh_-172 was not always sufficient to attenuate SOCE, and furthermore this inhibition was not reversed following 15–30 min of washout. This delayed effect suggests that CFTR_inh_-172 is unlikely to be a pore-blocker of SOC. The inhibition of SOCE may involve binding to Orai1 leading to allosteric changes in channel gating (similar to how CFTR_inh_-172 inhibits CFTR), or changes in the function and/or spatial localisation of the STIM1/Orai1 complex. Interestingly, CFTR itself has shown to be indirectly activated by Ca^2+^ (reviewed in Billet, Hanrahan [43]). Therefore, the inhibitory effects of CFTR_inh_-172 and GlyH-101 on CFTR-mediated anion transport may be partially attributed to the off-target downregulation of [Ca^2+^]_i_. Our recommendation would be to avoid extended pre-exposure to these inhibitors (i.e. more than 10 min to avoid potential off-target effects on SOCE complicating the interpretation of results.

Our findings also have a broader relevance to the putative role of CFTR in Ca^2+^ signalling in CF-affected cells. Previous reports have established that CF cells have enhanced SOCE [13, 44], and/or TRP-mediated Ca^2+^ entry [45], which has been linked to up-regulation of inflammatory pathways such as IL-8 secretion [13, 46] and a reduced ability to kill bacteria by neutrophils [47]. These findings have stimulated much interest in understanding the underlying molecular mechanisms for these effects of CFTR dysfunction on Ca^2+^ homeostasis. However, in some cases, conclusions have been based on the results using CFTR blockers [12–14]. Furthermore, the fact that CFTR inhibitors were capable of blocking SOCE, it is conceivable that putative specific SOC blockers could have off-target effects on CFTR which, as far as we are aware, has not to date been investigated.

In addition to off-target effects of CFTR inhibitors on Ca^2+^ transport, we also observed modulatory effects of the CFTR inhibitors on Na^+^ transport through ENaC. CFTR is often co-expressed with ENaC in epithelial tissues and the activity of both ion channels facilitates transepithelial ion secretion or absorption, respectively. This is best illustrated in airway epithelia, where CFTR-mediated Cl^−^ and HCO_3_^−^ secretion, together with ENaC-mediated Na^+^ absorption maintain the composition and volume of airway surface liquid [1]. Off-target effects of CFTR blockers on ENaC are therefore important when dissecting the contributions of these ion channels to transepithelial ion transport processes. To assess CFTR activity in airway epithelia, ENaC is often inhibited by amiloride to enhance the electrical gradient facilitating transepithelial anion secretion [48]. Any off-target effects of CFTR blockers on ENaC remain therefore undetectable using such protocols. ENaC is a heterotrimeric ion channel [32] which has two functional isoforms, depending on the channel’s subunit composition. ‘Classical’ αβγ-ENaCs are present in many sodium-absorbing epithelia [49], but the physiology of δβγ-ENaC remains unclear [50]. Using heterologous expression of human ENaC isoforms in *Xenopus* oocytes, we found that both CFTR blockers affected ENaC activity. While GlyH-101 strongly inhibited αβγ-ENaC and δβγ-ENaC, the effect of CFTR_inh_-172 was dependent on the ENaC subunit composition. While αβγ-ENaC was inhibited by CFTR_inh_-172, δβγ-ENaC was stimulated. Using molecular docking, we identified that both CFTR blockers, could bind to two putative binding sites in the extracellular domain of ENaC. The position of the first binding site agrees well with amiloride binding site identified in the related acid-sensing ion channel (ASIC1) [51]. Mutational studies of the ENaC WYKLHY (WYRFHY in rat) motif, which is in close proximity to the predicted first binding site, resulted in a loss of amiloride binding to ENaC [33]. In addition, mutagenesis of amino acids Gly-525, Gly-537 or Ser-583, which contribute to the second identified binding pocket for CFTR_inh_-172 and GlyH-101, were shown earlier to decrease channel blocking by amiloride [31, 52]. Experiments with these CFTR blockers in combination with mutagenesis of residues in the putative binding pockets, could provide functional evidence to support the structural predictions, which is worth exploring in the future. Currently, there is no structural information on δβγ-ENaC available, preventing us to hypothesise putative interaction sites between this channel isoform and the CFTR inhibitors. Nevertheless, while both ENaC isoforms are inhibited by amiloride, isoform-specific activation/inhibition by various compounds has been reported [53]. Structural differences between the ENaC isoforms likely account for the subunit-specific channel modulation by CFTR_inh_-172.

In conclusion, our data indicate that caution is needed when interpreting results using these two CFTR inhibitors, especially after prolonged exposure to the compounds, or when utilising calcium mobilising agonists that lead to activation of SOCE, and when investigating CFTR or ENaC function.

## Supplementary Information

Below is the link to the electronic supplementary material.

Supplementary file1 (DOCX 530 KB)

## Data Availability

The data that support the findings of this study are available from the corresponding author (m.a.gray@newcastle.ac.uk) upon reasonable request.
