# Supplementary material for: Pharmacological inhibitors of the cystic fibrosis transmembrane conductance regulator exert off-target effects on epithelial cation channels
Source: Pflugers Arch. 2022 Oct 7;475(2):167–79. doi: 10.1007/s00424-022-02758-9 (PMC9849171; doi:10.1007/s00424-022-02758-9)
Supplement: Supplementary file 1 — Supplementary file1 (DOCX 530 KB) [file 424_2022_2758_MOESM1_ESM.docx]

**Pharmacological inhibitors of the cystic fibrosis transmembrane conductance regulator exert off-target effects on epithelial cation channels**

JinHeng Lin, Sean M. Gettings, Khaoula Talbi, Rainer Schreiber, Michael J. Taggart, Matthias Preller, Karl Kunzelmann, Mike Althaus, Michael A. Gray.

**Supplementary Methods and Materials**

**Cell culture and transfection**

Calu-3 cells were cultured in Eagle’s minimum essential medium on 6.5mm semi-permeable inserts (Corning Costar® Transwells®, Sigma-Aldrich) until a confluent polarised monolayer was formed. Cells were used for experiments 10-14 days after seeding. Human embryonic kidney HEK293T cells were grown in DMEM low glucose medium supplemented with 10% FBS, 1% L-glutamine 200 mM (Invitrogen-Thermo Fisher Scientific).

*Transfection*

HEK293T cells were transfected using standard protocols for Lipofectamine 3000 with a bicistronic IRES plasmid vectors encoding CFTR and CD8. CFTR expressing cells were detected by binding of anti-CD8 labeled beads. All experiments were performed 72 h after transfection.

**Ussing chamber**

The inserts containing polarised Calu-3 cells were mounted onto the EasyMount Ussing Chamber Systems (EM-CSYS-6; Physiologic Instruments, San Diego, USA) according to manufacturer’s instructions; up to 6 inserts were mounted to perform parallel experiments. Cells were bathed in HEPES-buffered solution on both the apical and basolateral side, bubbled with 100% O_2_, and maintained at 37°C. Voltage (Ag-AgCl pellet electrode) and current (Ag wire electrode) electrodes were immersed in 3M KCl solution, topped with an agar salt bridge (4% agar in 3M KCl). One of each electrode was attached to each side of the Ussing chamber, and connected to a VCC MC6 multichannel clamp (Physiologic Instruments) to monitor transepithelial electrical resistance (TEER) and short-circuit current (I_SC_) across the epithelial membrane. The membranes were voltage-clamped to 0 mV, and real-time measurements of TEER and I_SC_ were recorded by injecting current pulses across the membrane every 10 s, through the Acquire and Analyze software (Physiologic Instruments). The I_SC_, representing the summation of ion currents across the membrane, was normalised to the membrane area of 0.33cm^2^.

**Whole Cell Patch Clamp**

Cells were patch-clamped after growing on coated glass coverslips for 2 days. Patch clamp experiments were performed in the fast whole-cell configuration. Patch pipettes had an input resistance of 3–6 MΩ, when filled with a solution containing (mM) KCl 30, K^+^-gluconate 95, NaH_2_PO_4_ 1.2, Na_2_HPO_4_ 4.8, EGTA 1, Ca^2+^-gluconate 0.758, MgCl_2_ 1.034, D-glucose 5, ATP 3. pH was 7.2, the Ca^2+^ activity was 0.1 μM. The access conductance was measured continuously and was 40–100 nS. Currents (voltage clamp) and voltages (current clamp) were recorded using a patch clamp amplifier (EPC 7, List Medical Electronics, Darmstadt, Germany), the LIH1600 interface and PULSE software (HEKA, Lambrecht, Germany) as well as Chart software (AD-Instruments, Spechbach, Germany). In regular intervals, membrane voltages (*V*c) were clamped in steps of 20 mV from −100 to +100 mV relative to resting potential. The experimental protocol consisted of activating CFTR currents with IBMX/Forskolin (I/F: 100/2 µM), then adding the inhibitor (CFTR Inh-172 (20µM) or GlyH-101 (10µM)) as soon as the I/F-induced current stabilized (after ~ 3 min). Data were stored continuously on a computer hard disc and were analyzed using PULSE software. Membrane conductance, *G*m, was calculated from the measured current (*I*) and *V*c values according to Ohm's law.


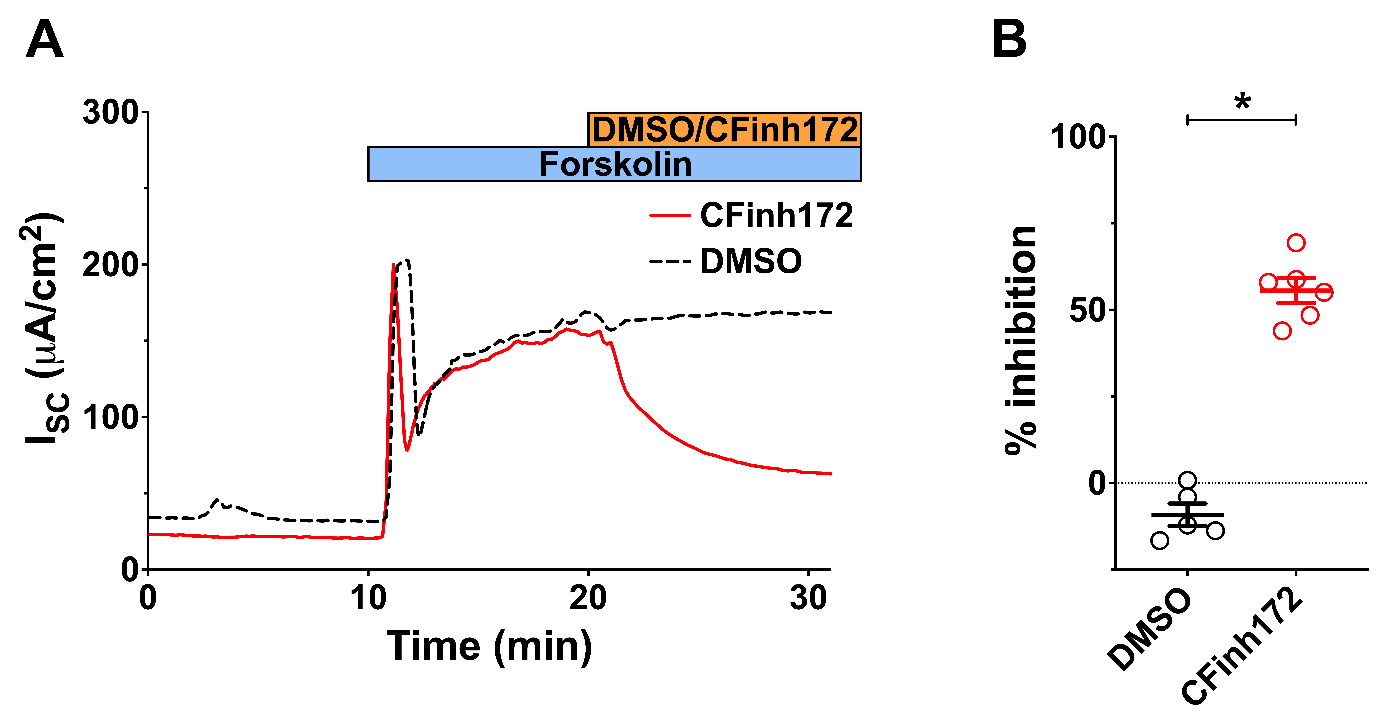


**Supplementary Figure 1. CFTR_inh_-172 inhibits forskolin-activated short circuit current in Calu-3 cells.** (A) Representative Ussing chamber traces tracking short circuit current (I_SC_) in polarised Calu-3 cells. Amiloride was present throughout each experiment to block ENaC. A Cl^-^ gradient of 134 mM basolateral to 0 mM apical was used to amplify Cl^-^ currents. (B) Summary of the percentage inhibition of forskolin-activated I_SC_ by DMSO or 20 μM CFTR_inh_-172 (CFinh172). Unpaired t-test was performed between the two treatment groups. n = 5-6. Data presented as mean ± SEM. A p-value of < 0.05 was accepted as statistically significant.


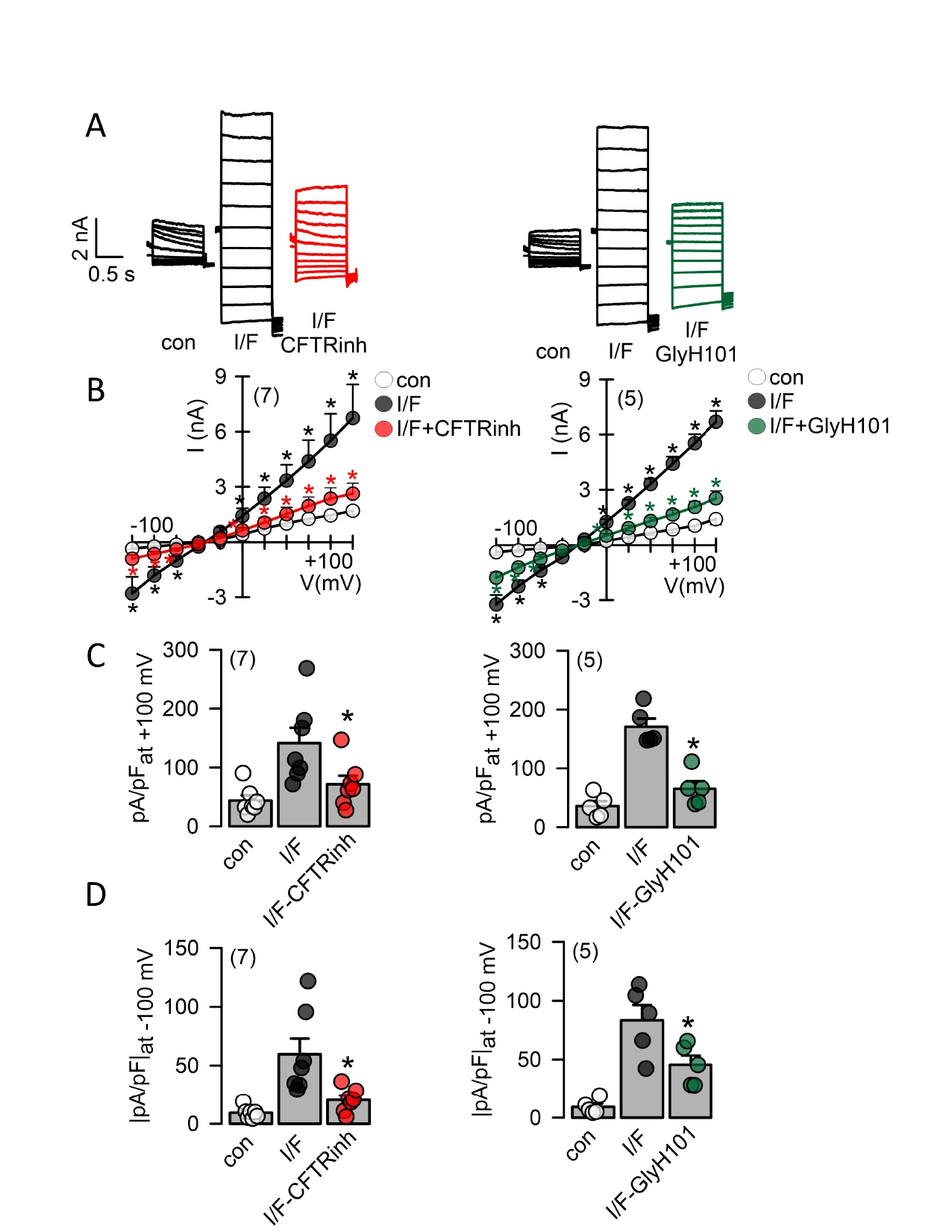


**Supplementary Figure 2. CFTR_inh_-172 and GlyH-101 inhibit forskolin-activated whole cell current in HEK293T cells.** (A) Original whole cell patch clamp recordings in HEK293T cells overexpressing CFTR. Currents were obtained under basal conditions (con), after stimulation with IBMX/Forskolin (I/F, 100 µM/ 2 µM) and after inhibition with CFTR_inh_-172 (20 µM) or GlyH-101 (10 µM) in the presence of I/F. (B) I/V curves from the currents shown in A. (C) Summaries of the current densities at +100 mV for the currents summarized in I/V curves shown in B. (D) Summaries of the current densities at -100 mV for the currents summarized in I/V curves shown in B. (Number of cells). Data are shown as mean values ± SEM. *Significant difference when compared to I/F stimulation, paired *t* test. A p-value of < 0.05 was accepted as statistically significant.
